# Validation of accuracy of SVM-based fall detection system using real-world fall and non-fall datasets

**DOI:** 10.1371/journal.pone.0180318

**Published:** 2017-07-05

**Authors:** Omar Aziz, Jochen Klenk, Lars Schwickert, Lorenzo Chiari, Clemens Becker, Edward J. Park, Greg Mori, Stephen N. Robinovitch

**Affiliations:** 1 Injury Prevention and Mobility Laboratory, Simon Fraser University, Burnaby, British Columbia, Canada; 2 School of Engineering Science, Simon Fraser University, Burnaby, British Columbia, Canada; 3 School of Mechatronic Systems Engineering, Simon Fraser University, Surrey, British Columbia, Canada; 4 Department of Clinical Gerontology, Robert-Bosch-Hospital, Stuttgart, Germany; 5 Institute of Epidemiology and Medical Biometry, Ulm University, Ulm, Germany; 6 Department of Electrical, Electronic, and Information Engineering “Guglielmo Marconi”, University of Bologna, Bologna, Italy; 7 School of Computing Science, Simon Fraser University, Burnaby, British Columbia, Canada; 8 Department of Biomedical Physiology and Kinesiology, Simon Fraser University, Burnaby, British Columbia, Canada; University of Illinois at Urbana-Champaign, UNITED STATES

## Abstract

Falls are a major cause of injuries and deaths in older adults. Even when no injury occurs, about half of all older adults who fall are unable to get up without assistance. The extended period of lying on the floor often leads to medical complications, including muscle damage, dehydration, anxiety and fear of falling. Wearable sensor systems incorporating accelerometers and/or gyroscopes are designed to prevent long lies by automatically detecting and alerting care providers to the occurrence of a fall. Research groups have reported up to 100% accuracy in detecting falls in experimental settings. However, there is a lack of studies examining accuracy in the real-world setting. In this study, we examined the accuracy of a fall detection system based on real-world fall and non-fall data sets. Five young adults and 19 older adults went about their daily activities while wearing tri-axial accelerometers. Older adults experienced 10 unanticipated falls during the data collection. Approximately 400 hours of activities of daily living were recorded. We employed a machine learning algorithm, Support Vector Machine (SVM) classifier, to identify falls and non-fall events. We found that our system was able to detect 8 out of the 10 falls in older adults using signals from a single accelerometer (waist or sternum). Furthermore, our system did not report any false alarm during approximately 28.5 hours of recorded data from young adults. However, with older adults, the false positive rate among individuals ranged from 0 to 0.3 false alarms per hour. While our system showed higher fall detection and substantially lower false positive rate than the existing fall detection systems, there is a need for continuous efforts to collect real-world data within the target population to perform fall validation studies for fall detection systems on bigger real-world fall and non-fall datasets.

## Introduction

Falls are the number one cause of injuries and injury-related deaths in older adults [1, 2]. About half of older adults who fall are unable to get up without assistance, even when no injury occurs [3, 4]. The ensuing “long lie” on the floor often leads to dehydration, muscle damage, and fear of future falls [5, 6].

Automatic fall detection systems based on wearable accelerometers and/or gyroscopes can reduce the risk for long lies after a fall, by detecting the fall and quickly alerting care providers of the event [7–12]. The challenge is to develop devices that can detect falls with high accuracy, while being sufficiently unobtrusive and comfortable to meet with high user acceptance.

While the technology is becoming more available to meet these demands, the lack of evidence on the accuracy of automatic fall detection devices in older adults is a major barrier to the refinement and uptake of this technology. The accuracy of automatic fall detection systems has traditionally been evaluated in laboratory simulations where young adults fall onto padded mattresses. For example, Diaz et al. [13] found that a system incorporating triaxial accelerometers at the waist and a threshold-based fall detection algorithm was able to distinguish falls from activities of daily living (ADLs) with 100% sensitivity and 92.5% specificity. Kangas [14, 15] found that a threshold-based algorithm and tri-axial accelerometers at the waist or head provided up to 98% sensitivity in detecting falls. Bourke et al. [11] considered thresholds for velocity in the pre-impact phase, acceleration during impact, and body angle post-impact, and observed 100% sensitivity and 100% specificity in fall detection.

The key limitation of this approach is that young adults falling in a laboratory setting may not accurately simulate the movement patterns that are typical of falls in older adults. Accordingly, fall detection algorithms that are developed based on sensor data from simulated falls in young adults would lack generalizability and external validity for distinguishing falls in the target population of older adults. This concern was highlighted by Klenk and colleagues [16], who compared acceleration signals from simulated falls by young adults to real-world backward falls experienced by older adults, and reported substantially higher variations in acceleration for falls in older adults. The authors surmised that the differences were due in part to the use in real-world falls (and absence in simulated falls) of compensatory strategies aimed at recovering balance or avoiding injury. In real-world falls, people use compensatory strategies to prevent an impact because their intention is not to fall. These strategies may be absent in simulated falls, where the intent is to fall, and where participants are aware they are falling onto a soft mattress.

These concerns highlight the need for testing automatic fall detection devices in real-world settings. We are aware of only two studies that have evaluated the accuracy of wearable sensor systems in detecting falls in the real-world settings. Bagalà et al. [17] evaluated 13 threshold-based fall detection methods based on accelerometer data from 29 real-world falls in older adults, and found considerably lower sensitivity and specificity than the values reported for the simulated falls. The average sensitivity of the 13 methods was 57.0% (SD = 27.3%; maximum value = 82.8%), and the number of false alarms generated during 1-day of monitoring ranged from 3 to 85. More recently, Lipsitz and colleagues [18] evaluated an investigational fall detection device produced by Philips (Amsterdam, Netherlands) on a real-world dataset. This pendant-style fall detection device was tested on older adults living in nursing homes who were identified with higher risks of falls. During 6 months of continuous monitoring, 89 falls occurred among the older adults, and the device detected only 17 of them. Moreover, the device labeled 128 instances as fall events out of which, 111 were false positives.

The problem of false positives and false negatives is unfortunately not solved adequately through an onboard button, which a wearer can manually press to activate or deactivate an alarm, since this approach assumes the person is conscious and capable of pressing the button [19–21]. These criteria are often not met after a fall, especially among older adults with physical and/ or cognitive impairment. A cohort study by Fleming and Brayne [22] found that nearly 80% of older adults who had emergency response systems available to them did not use their alarm system to call for help after experiencing a fall.

In an effort to improve the accuracy of automatic fall detection systems, we recently showed that machine learning approaches for interpreting signals from waist-mounted tri-axial accelerometers are at least as accurate as threshold-based techniques in distinguishing simulated fall from non-fall datasets [23]. Moreover, we found that a Support Vector Machine (SVM) provided the best fall classification accuracy among the five machine learning algorithms we tested (Logistic Regression, Naïve Bayes, Decision Trees, K-Nearest Neighbor, and SVM). Our findings were supported by Liu et al. [24] who also found that an SVM fall detection model, using daily activity data from a waist-mounted triaxial accelerometer, provided promising rates of false positives (0.18 per hour for older adults), although the algorithm was not evaluated for sensitivity (since no falls occurred over the period of monitoring).

Against this background, our goal in the current study was to test the accuracy of our SVM-based machine learning fall detection algorithm with signals from tri-axial accelerometers recorded from young and older adults during real-world falls and daily activities. We designed our SVM algorithm using data from simulated falls and non-fall trials acquired in the laboratory with young adults. We then tested the system with real-world data from young healthy volunteers, and older adults in long-term-care and acute care settings. We describe the accuracy of our SVM classifier in terms of sensitivity, specificity, and rates of false positives. We hypothesized that our algorithm would perform at least as well as the best threshold-based algorithm evaluated by Bagalà et al. [17] using real-world fall and ADL data (83% sensitivity and 97% specificity).

## Material and methods

### 1. Real-world measures with young adults

Five young and healthy volunteers, ranging in age from 26 to 35 years (mean = 30.8 years, SD = 4.1 years) wore an array of seven tri-axial accelerometers (range ±6 g, Opal model, APDM Inc., Portland, OR) mounted bilaterally on the ankles and thighs, and at the waist, sternum and head. Approximately six daytime hours of time-stamped data were recorded from each participant who went about their normal everyday activities while wearing the sensors. A total of 28 hours and 38 minutes of data were recorded. None of the participants reported any fall during that time. The data collection was approved by the Research Ethics Committee at Simon Fraser University and all participants provided informed written consent.

### 2. Real-world measures with older adults at LTC

Nine older adults residing at New Vista long-term care (LTC) facility in Burnaby, British Columbia were participants in sensor data collection. Participants ranged in age from 76 to 94 years (mean = 87.4 years, SD = 6.1 years). Each participant had experienced at least one fall (based on fall incident reports) during the past 12 months, and was able to transfer independently to and from a bed or chair, and walk independently (with or without a walker or cane). Data were recorded from four tri-axial accelerometers (range ±6 g, Opal model, APDM Inc., Portland, OR) worn bilaterally on the ankles, and at the waist and sternum. A member of the research team placed the sensors on the participant every morning and removed them for recharging and data download at night before the individual went to bed. A researcher also stayed at the facility from morning until night, checking on the resident every 30 minutes and logging their activities.

**Fig 1:**
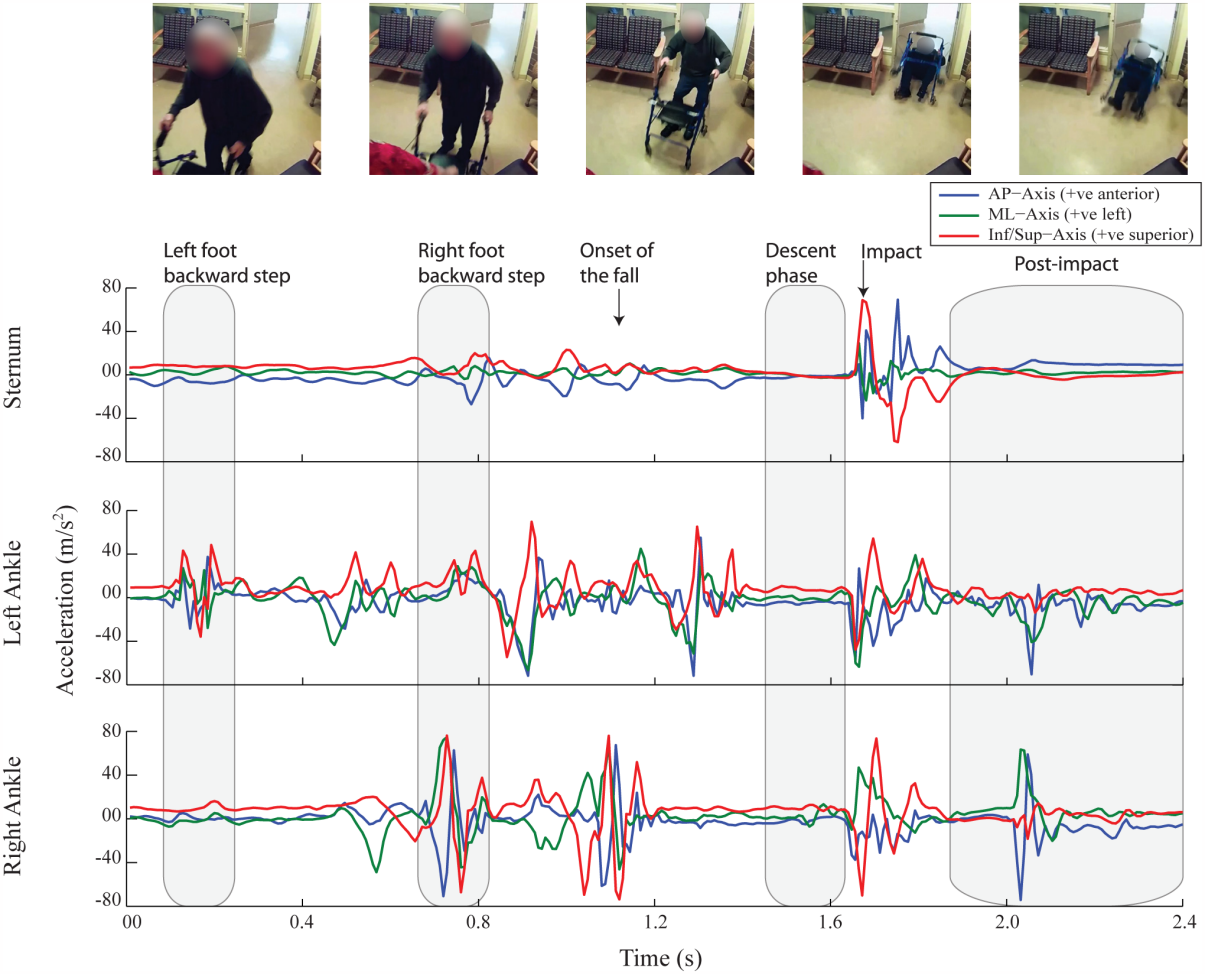
Acceleration traces from a real-world backward fall. Sternum, left ankle and right ankle mounted tri-axial accelerometer signals in anterior/ posterior (AP), medial/ lateral (ML) and inferior/ superior (Inf/Sup) directions from a real-world backward fall recorded at New Vista LTC.

A total of 214 hours of time-stamped sensor data were recorded from the nine participants over two months. During this time, one unintentional fall was recorded. The LTC facility was equipped with a network of 48 surveillance cameras installed in common areas for residents’ safety. Members of our research team communicated daily with care providers and retrieved fall video footage, if any. Video footage of the fall occurring during sensor data collection was retrieved (Fig 1). The experimental protocol was approved by the Research Ethics Committee at Simon Fraser University. In addition, all participants provided informed written consent for data collection using video cameras and wearable sensors.

### 3. Real-world measures with older adults at acute care

Starting January 2012, several hundred older adults belonging to community, geriatrics rehabilitation center of Robert Bosch Krankenhaus (RBK), nursing home and assistive living centers were recruited to participate in a study to record their physical activities and unintentional falls using wearable sensors. In the current study, we utilized fall and non-fall data from 10 older inpatients and outpatients from the Geriatrics Department at RBK in Stuttgart, Germany, who ranged in age between 56 and 75 years (mean = 67.3 years, SD = 6.4 years), and had diagnosed progressive supranuclear palsy (PSP). Data were recorded from each participant using a tri-axial accelerometer (range ±2 g, Dynaport MiniMod, McRoberts, The Hague, NL) mounted at the posterior aspect of the waist. From the ten patients, a total of 172 hours and 12 minutes of time-stamped sensor data were recorded. During this time, 9 real-world falls were reported. Up until 2013, the occurrences of falls and their corresponding times were reported by the patients and/or their proxies, and were verified by research engineers through visual inspection of sensor signals at the reported times. Ethical approval for sensor data collection was provided by the Ethical Committee of the University of Tübingen (495/2012BO2) and the data protection office of the federal state of Baden-Württemberg, Germany (T 1500/231).

## Data analysis

Data analysis focused on determining the accuracy of our SVM classifier, trained on laboratory data, in distinguishing falls from normal ADLs based on real-world fall and non-fall data.

### 1. Model training using laboratory data

**Fig 2:**
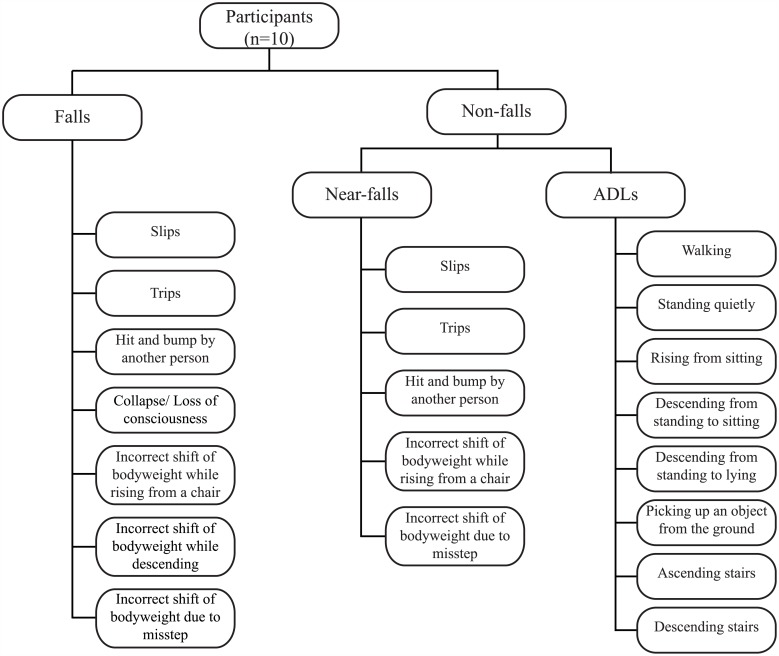
Experiment protocol. The experiment protocol indicating the 7 types of falls, 5 near- falls, and 8 activities of daily living (ADLs) simulated by each participant. Ten participants performed three repeated trials for each category.

An SVM model using a Radial Basis Function (RBF) kernel was trained [25] using data from 10 young adults. Participants wore an array of seven sensors (range ±6 g, Opal model, APDM Inc., Portland, OR) during laboratory experiments that simulated seven types of falls, selected to represent the most common falling scenarios (causes of imbalance and activities during falling) among older adults in long-term care [26]. Data were also acquired for a variety of near-falls and while the participant performed activities of daily living (ADLs). The list of falls, near-falls and ADLs simulated in the laboratory experiment is shown in Fig 2, and the details of the experimental protocol and how the falls and non-fall trials were simulated can be found in Aziz et al. [23].

3D acceleration signals recorded from fall and non-fall trials were used to calculate means and variances for each of the X, Y and Z axes over 2.5 s time window which resulted in a 6 dimension ‘feature vector.’ Furthermore, the two RBF kernel parameters *C* and *γ* were selected by a grid-search with exponential growing sequence (*i*. *e*. *C* ∈ {2^−5^, 2^−4^, …, 2^14^, 2^15^} *and γ ∈* {2^−15^, 2^−14^, …, 2^2^, 2^3^}). Each combination of parameters was tested with a 10-fold cross-validation, and the parameter combination with the best cross-validation accuracy was selected. The final model, used for classifying the real-world data, was then trained on the entire fall and non-fall data set from the 10 young adults.

### 2. Model testing using real-world data

The test data were obtained by calculating features (means and variances) of 3D acceleration signals recorded from 2.5-s consecutive time windows that overlapped by 1.5-s. In order to standardize the range of the features, they were normalized between 0 and 1 during both model training and testing. Furthermore, to avoid multiple predictions of the same fall event, each time the classifier predicted a fall, a delay of 30 seconds was inserted before restarting the algorithm to consider the possibility of a subsequent fall.

Since the anatomical location of the sensors varied between the three participant sub-groups (young adults and older adults in Burnaby, Canada; and older adults in Stuttgart, Germany), we tested the fall detection accuracy of the trained SVM on each group separately. With young adults, data from each of the head, sternum and waist sensors were tested for fall detection. Each sensor location was tested using a model trained from data recorded from the sensor worn at the same location during laboratory simulations. With residents at LTC facilities, the fall detection model was tested using the sternum accelerometer, since logistical issues impaired the collection of continuous data from the waist (several participants complained of discomfort during sitting and toileting, in which case care staff removed the waist sensors). With patients at RBK Geriatrics Department, waist mounted accelerometer data were tested for fall detection. The sensor was placed at the posterior aspect of the waist (likely contributing to greater comfort and compliance in wearing the device).

In evaluating each fall detection model, we focused on three measures of performance:

Sensitivity—the capacity of the system to detect falls and corresponds to the ratio of true positives to the total number of falls:

$$Sensitivity=\frac{True\;Positive}{True\;Positive+False\;Negative}$$ (1)

Specificity–the capacity of the system to avoid false alarms and corresponds to the ratio of true negatives to the total number of discarded trials:

$$Specificity=\frac{True\;Negative}{True\;Negative+False\;Positive}$$ (2)

Finally, the false positive rate—the number of false alarms per hour and corresponds to the ratio of the total number of false positives divided by the total recording time in hours:

$$False\;positive\;rate=\frac{False\;Positive}{ADL\;Time\;(in\;hours)}$$ (3)

All data analysis was performed using MATLAB (R2014a, The MathWorks Inc.).

## Results

Our fall detection system showed perfect accuracy with all five young participants (Table 1). No false alarms were generated in the 28 hours and 38 minutes of real-world recorded data. All three sensor-locations (head, sternum and waist) provided 100% specificity.

**Table 1:** Fall detection performance indicators calculated from five young adults using support vector machine classifier.

Participants	Recorded duration (hh:mm:ss)	Head	False Pos. (n)	False Pos. rate (per hour)	Sternum	False Pos. (n)	False Pos. rate (per hour)	Waist	False Pos. (n)	False Pos. rate (per hour)
		Spec. (%)			Spec. (%)			Spec. (%)		
**Participant 1**	6:15:10	100	00	00	100	00	00	100	00	00
**Participant 2**	5:09:51	100	00	00	100	00	00	100	00	00
**Participant 3**	4:57:57	100	00	00	100	00	00	100	00	00
**Participant 4**	5:52:18	100	00	00	100	00	00	100	00	00
**Participant 5**	6:23:01	100	00	00	100	00	00	100	00	00
**Total**	28:38:17	100	00	00	100	00	00	100	00	00

Notes: Spec. = Specificity; False Pos. = False Positive.

The system successfully detected the only fall occurred among the 9 residents of the Burnaby LTC facility during 214 hours of recorded sensor data (Table 2). During this time, a total of 10 false alarms were generated which corresponded to 0.05 false alarms per hour. At the individual level, no false alarms were generated with 3 residents, one alarm occurred in each of four residents, and two residents had 3 and 4 false alarms during approximately 17.5 hours and 14.5 hours of data recording respectively. The highest false positive rate was 0.27 for resident number 4.

**Table 2:** Fall detection performance indicators calculated using support vector machine classifier from nine older adults residing in New Vista LTC facility.

Participants	Recorded duration (hh:mm:ss)	Falls (n)	Sens. (%)	Spec. (%)	False Neg. (n)	False Pos. (n)	False Pos. rate (per hr)
Resident 1	83:37:11	01	100.00	100.00	00	00	0.00
Resident 2	28:34:43	00	n/a	99.99	n/a	01	0.03
Resident 3	17:38:28	00	n/a	99.99	n/a	02	0.11
Resident 4	14:37:58	00	n/a	99.99	n/a	04	0.27
Resident 5	5:32:54	00	n/a	100.00	n/a	00	0.00
Resident 6	26:25:41	00	n/a	99.99	n/a	01	0.04
Resident 7	18:15:20	00	n/a	100.00	n/a	00	0.00
Resident 8	12:06:01	00	n/a	99.99	n/a	01	0.08
Resident 9	7:11:59	00	n/a	99.99	n/a	01	0.14
Total	214:00:15	01	100	99.99	00	10	0.05

Notes: Sens. = Sensitivity; Spec. = Specificity; False Neg. = False Negative; False Pos. = False Positive.

For the RBK Geriatrics Department patients, the fall detection system successfully identified 7 out of 9 falls during 178 hours of recorded sensor data (Table 3). A total of 26 false positives were reported at the rate of 0.15 false alarms per hour. At the individual level, 4 or fewer false alarms were generated from 8 patients with half of those patients not having any false alarm. Two patients had 6 and 7 false alarms during approximately similar data recording time of 23.5 hours. The highest false positive rate was recorded as 0.3 with patient number 1. Furthermore, the 2 falls missed by the algorithms were from the same patient (patient number 7).

**Table 3:** Fall detection performance indicators calculated from 10 patients at RBK geriatrics department using support vector machine classifier.

Participants	Recorded duration (hh:mm:ss)	Reported falls (n)	Sens. (%)	Spec. (%)	False Neg. (n)	False Pos.(n)	False Pos. rate (per hr)
Patient 1	23:33:41	02	100	99.99	00	07	0.30
Patient 2	23:30:10	00	n/a	99.99	n/a	04	0.17
Patient 3	23:43:57	01	100	99.99	00	06	0.25
Patient 4	14:03:20	01	100	100.00	00	00	0.00
Patient 5	13:50:40	01	100	100.00	00	00	0.00
Patient 6	23:26:43	02	100	99.99	00	03	0.13
Patient 7	13:35:48	02	00	99.99	02	04	0.29
Patient 8	13:35:28	00	n/a	100.00	n/a	00	0.00
Patient 9	11:14:05	00	n/a	100.00	n/a	00	0.00
Patient 10	11:38:12	00	n/a	99.99	n/a	02	0.17
Total	172:12: 04	09	78	99.99	2	26	0.15

Notes: Sens. = RBK = Robert Bosch Krankenhaus; Sensitivity; Spec. = Specificity; False Neg. = False Negative; False Pos. = False Positive.

## Discussion

In the current study, we examined the accuracy of an accelerometer-based automatic fall detection algorithm in distinguishing falls in older adults. The algorithm incorporated an SVM machine learning algorithm that was trained using laboratory-based falls and non-fall data, and tested with sensor data acquired from real-world falls and during daily activities by older adults. We found that, with 3D acceleration data from a single sensor, our algorithm showed 80% sensitivity (8 out of 10 real-world falls were successfully detected) and 99.9% specificity (false positive rates from 0.05 to 0.15 false alarms per hour depending on the older adult dataset of approximately 214 hours and 172 hours of ADLs). This is comparable to, if not better than the best performing algorithm reported by Bagalà et al. [17] which showed 83% sensitivity (23 out of 29 falls were successfully detected) and 97% specificity (false positive rate of 0.21 false alarms per hour on the older adult dataset of approximately 24 hours of ADLs).

Our approach diverged from traditional threshold-based fall detection methods to include advanced machine-learning techniques, and this may account in part for the improved accuracy. Another likely source of the improvement is the data source used in training our fall detection algorithm. We designed our laboratory falling simulations to include the most common circumstances of real-world falls in older adults. Previous investigators have developed and evaluated their algorithms based on data from young subjects falling in the forward, backward and/ or sideways direction from a static, standing position. However, recent findings from our laboratory [26] have shown that approximately 48% of falls among older adults residing in long-term-care facilities occur while walking and 86% of the falls are collectively due to: (i) incorrect shift of bodyweight (41%), (ii) trip (21%), (iii) hit/ bump (11%), (iv) collapse/ loss of conscious (10%) and (v) slip (3%). These falling scenarios are substantially different from the ones employed in the past for simulating falls in the laboratory environment. For each of these categories, we used video clips of actual falls among older adults to train young subjects to imitate the real-world fall. Furthermore, we incorporated near-falls (imbalance episodes followed by successful balance recovery) along with ADL trials to improve the quality of our non-fall dataset. We previously showed [23] that near-falls are often accompanied by high accelerations, and may produce false alarms if not included in model training.

While our system performance appears to be comparable with the best threshold-based systems, further improvement is required to reduce the rate of false positives. The occurrence of false positives could have been due to the relatively similar number of non-fall trials and fall trials simulated in our laboratory experiments. The algorithm was trained with 210 falls and 390 non-fall trials. In everyday life, even among frail older adults, the proportion of time spent in performing normal activities greatly outweighs the time related to falls. Ideally, this distribution should be reflected in the data used for training a classification model. It is also possible that fall events exist in the data which were not reported (i.e., some false positives were actual falls) for participants recruited through the RBK Geriatrics Department, whose falls were self-reported. We expect the risk of missed falls to be lower for participants in LTC, where nursing staff are required to complete incident reports documenting all falls, and it is unlikely that falls are not identified and reported by care staff. Finally, as the real-world fall and non-fall datasets grow (e.g. EU-based FARSEEING database), it would be possible in the future to develop fall detection algorithms directly using the real-world data. This would potentially improve the accuracy of fall detection algorithm in general, and in particular those algorithms that utilize machine learning approaches.

In summary, through acceleration data obtained from simulated falls by imitating the most common causes of falls among older adults in long-term care facilities, we show that a robust fall detection algorithm can be designed for distinguishing real-world data into falls and non-falls. Finally, access to a robust data set is critical to the development of more accurate fall detection algorithms. Previous studies generally describe the tests performed and the results obtained, but sensor raw data are usually not publicly made available. The collection of real-world fall data is difficult and time-consuming, yet critical to algorithm testing. This points towards the need for shared databases of real-world fall and ADL data, available to the scientific community to design and test their fall detection algorithms.
